# Impacts of Conformational Geometries in Fluorinated Alkanes

**DOI:** 10.1038/srep31382

**Published:** 2016-08-16

**Authors:** Tim Brandenburg, Ronny Golnak, Masanari Nagasaka, Kaan Atak, Sreeju Sreekantan Nair Lalithambika, Nobuhiro Kosugi, Emad F. Aziz

**Affiliations:** 1 Institute of Methods for Material Development, Helmholtz-Zentrum Berlin für Materialien und Energie, Albert-Einstein-Straße 15, 12489 Berlin, Germany; 2 Department of Physics, Freie Universität Berlin, Arnimallee 14, 14195 Berlin, Germany; 3 Institute for Molecular Science, Myodaiji, Okazaki 444-8585, Japan

## Abstract

Research of blood substitute formulations and their base materials is of high scientific interest. Especially fluorinated microemulsions based on perfluorocarbons, with their interesting chemical properties, offer opportunities for applications in biomedicine and physical chemistry. In this work, carbon K-edge absorption spectra of liquid perfluoroalkanes and their parent hydrocarbons are presented and compared. Based on soft X-ray absorption, a comprehensive picture of the electronic structure is provided with the aid of time dependent density functional theory. We have observed that conformational geometries mainly influence the chemical and electronic interactions in the presented liquid materials, leading to a direct association of conformational geometries to the dissolving capacity of the presented perfluorocarbons with other solvents like water and possibly gases like oxygen.

Research in blood substitute formulations is gaining more attention in the scientific community in recent years^1^,^2^,^3^,^4^,^5^,^6^,^7^,^8^,^9^ as more pharmaceutical companies start clinical trials on various artificial blood approaches^7^,^10^,^11^,^12^. In general, artificial blood consists of emulsions of different liquid or protein compounds to increase oxygen solubility and transport capabilities as well as to decrease toxicity to biological tissue^7^,^10^. Two of the more promising approaches center themselves around hemoglobin-based carriers^11^ and fluorinated microemulsions^6^,^9^. Despite setbacks regarding clinical tests and medicinal approval of recent formulations^5^,^13^, an emulsion containing perfluorodecalin named Fluosol^12^ was successfully approved by the U.S. Food and Drug Administration in 1989 (New Drug Application N860909).

The main oxygen carrier in Fluosol, perfluorodecalin, is part of the family of perfluorocarbons. They have a wide range of applications ranging from tissue oxygenation^1^,^14^,^15^ to post-operative treatment^16^,^17^. Their wide range of extraordinary properties - high density, high viscosity, high biological and chemical inertness, high gas solubility^18^,^19^ - offer opportunities for applications in biomedicine and physical chemistry^20^,^21^, therefore leading to a high interest in scientific development^2^,^18^,^19^,^22^,^23^,^24^,^25^.

An inherent feature to all perfluorocarbons is the so called “perfluoro effect”, which describes the energy shifts of the spectral features due to the stabilization effect of fluorine in the fluorination process^24^,^25^,^26^,^27^. The magnitude of the energy shift can subsequently be used for a classification of a molecular orbital (MO) to either bear σ- or π-character^24^,^25^ delivering an experimental method for the orbital classification. Some experimental studies based on photoelectron and X-ray absorption (XA) spectroscopy have already been performed^24^,^25^,^26^,^27^,^28^, but the development of more complex theoretical models^29^ and new experimental techniques^30^,^31^,^32^ gives opportunities for further investigation of perfluorocarbons.

In liquid emulsions several effects need to be accounted for. Two of the more prominent are the conformation effect^33^ and the van der Waals force^34^. The conformation effect affects peak broadenings and is a result of excited orbital interactions with surrounding molecules of different conformations^33^,^35^. In the case of nonpolar systems, the acting van der Waals force is the London dispersion force, that is, an attractive force resulting from induced dipoles^34^. The objective of the present work is to provide experimental and theoretical information on the electronic structure of liquid fluoroalkanes and their respective hydrocarbons, which are subject to weak van der Waals forces and have a high amount of different conformational geometries^18^,^36^, through element specific XA spectroscopy. A discussion of experimental XA and theoretically calculated spectra is presented revealing a strong involvement of the conformation effect with the local electronic structure and relative inertness of liquid fluoroalkanes. We additionally expose a direct association of different conformational geometries to the dissolving capacity of the presented perfluorocarbons with other solvents like water and possibly gases like oxygen.

## Results

**Figure 1:**
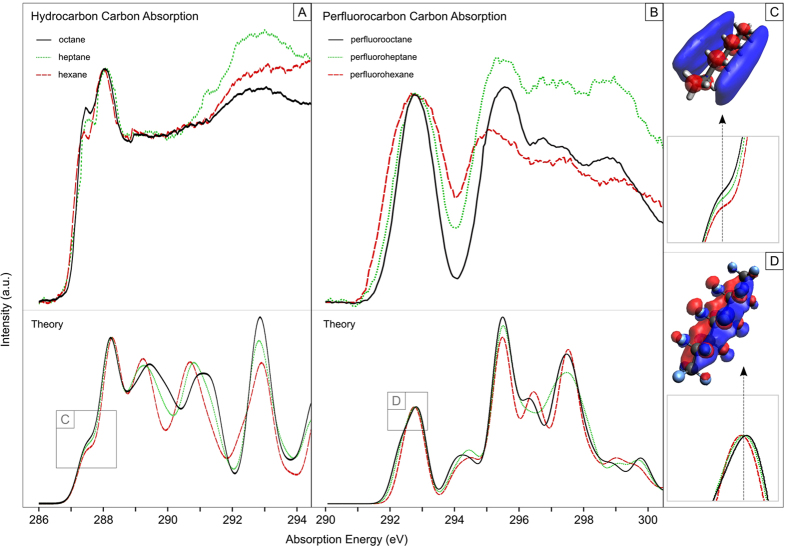
Experimental and theoretical carbon K-edge X-ray absorption data for chain-shaped hydrocarbons (**A**) and their respective perfluorocarbons (**B**). The insets (**C**,**D**) represent the main areas of change in the experimental spectra and illustrate the molecular orbitals for the corresponding transitions.

The experimental carbon K-edge XA spectrum of the hydrocarbons (Fig. 1A) reveals a striking similarity between the different chain-shaped molecules hexane, heptane and octane. The presence of a pre-peak at 287.5 eV, the main feature around 288.05 eV and a broad band at 292.8 eV can be identified in all cases. By employing theoretical calculations based on the DFT algorithm for geometry optimizations and TD-DFT for spectral calculations on single molecules, the general spectral shape of the XA spectra can be reproduced (Fig. 1A). Based on the calculations and previously proposed assignments^37^, the broad band feature around 292.8 eV is assigned to σ^*^(C-C) shape resonances. The calculated bond lengths of the hydrocarbons (1.53 Å uniformly) further support this assignment, due to the correlation of σ^*^ resonance positions with the bond length of hydrocarbons^38^. The main feature at 288.05 eV exhibits, in contrast to the conclusions on the gas phase drawn by Hitchcock *et al*.^37^, no significant changes between the different hydrocarbons. Hitchcock *et al*. observed a decrease in peak area and height as the amount of (CH_2_)-chains increase. However, the provided data^37^ shows a significantly smaller decrease upon reaching hexane and our theoretical calculations give no hints of changes in the major feature. Taking the presented data in this work into account, we conclude a smaller influence of the peak area and height decrease effect as the amount of (CH_2_)-chains exceeds four. We also observe an intensity increase and energy shift (100 meV) in the pre-peak feature as the number of (CH_2_)-chains increases, which is in accordance with the data provided by Hitchcock *et al*. for chains with one to four CH_2_^37^. The intensity increase can be explained through a higher cross-section for this particular transition as the amount of (CH_2_) carbons is increased, which is also confirmed by the simulated spectra (Fig. 1C), albeit no hint to an energy shift is observed and the intensity increase is underestimated. Small energy shifts can result from intermolecular interactions^35^,^39^. The theory doesn’t include intermolecular interactions, but chain-shaped hydrocarbons are molecules, which are confined in the vicinity of each other in liquid phase due to strong intermolecular forces^40^. Here, the dominant forces are London dispersion forces^36^,^41^,^42^, which are an attractive force resulting from temporary dipoles induced by interactions of electrons in two adjacent molecules. London forces become stronger as the molecule grows larger due to a higher contact interaction from the increased surface area^41^,^42^. Strong dispersed electron clouds over the surface further amplify this effect. The unoccupied orbital correlated to this particular transition is provided in Fig. 1C and exhibits a strong distribution over the intermolecular contact surface; however, this effect is applied not only to the ground state but also to the core excited state, resulting in minor effects in the XA peak shift and width.

Figure 1B shows the experimental carbon K-edge XA spectra of the perfluorocarbons perfluorohexane (PFHex), perfluoroheptane (PFHep) and perfluorooctane (PFO) and the corresponding theoretical calculations. Theory predicts the spectral shape reasonably well, with a strong resonance at 292.8 eV, a dip around 294.1 eV and several shape resonances above 295 eV. According to the calculations we can assign the resonances above 295 eV uniformly as σ^*^(C-C) shape resonances for all perfluorocarbons. The main resonance at 292.8 eV exhibits a decrease in full-width at half maximum (FWHM) as the amount of (CF_2_)-chains increases. The FWHM decreases from 2 eV (PFHex) over 1.5 eV (PFHep) down to 1.2 eV (PFO). As the FWHM decrease is not depicted in the theoretical predictions, the most likely cause is an effect not incorporated in the calculations, like intermolecular interactions. In the case of isolated atomic and molecular systems, the width of a resonance is determined by the inverse of the lifetime of the final state^43^. The life time broadening is not so much affected by intermolecular interactions in the case of interacting systems such as clusters and liquids. Instead, different molecular conformations can affect peak broadenings due to the exchange interaction of excited electrons with surrounding molecules of different conformations^33^,^35^. This so called “conformation effect” can be exhibited if the excited orbital is of delocalized character, as delocalized orbitals are more sensitive to the surrounding, and gains influence if the amount of different molecular conformations in a liquid is high. Perfluorocarbons are known for having a high amount of slightly distorted molecular geometries, due to the substitution of hydrogen to the electronegative and bigger fluorine^18^, which subsequently results in a high amount of different conformational geometries. In case of perfluoroalkanes the amount of geometries is also directly affected by the length of the chain and should lead to a higher peak broadening for PFHex compared to PFO, due to the conformation effect and the higher amount of different conformational geometries. The feature at 292.8 eV can mainly be attributed to π_z_(C-C) resonances (see Fig. 1D), which are of delocalized character. As mentioned above, different intermolecular interactions of an excited orbital of delocalized character with the surroundings results in an influence on the peak broadening. As the amount of (CF_2_)-carbons increases, the size of the molecule and accordingly also the size of the MO increases (see Fig. 1D and supplementary material), resulting in more delocalized orbitals and more conformational geometries. This directly leads to a higher peak broadening for PFHex compared to PFO due to the more delocalized orbital and the intermolecular conformation effect. Subsequently, PFHex has the widest FWHM from the compared perfluorocarbons and PFO the narrowest as a direct outcome from the difference in conformational geometries. Additionally, an investigation of different dimer, trimer and more complex structures would improve the understanding of the impact of conformational geometries in the given materials. Subsequently, molecular dynamics investigations of different conformations are recommended to extend the analysis.

Within the theory of the frontier orbital model the lowest unoccupied molecular orbitals (LUMO) play a major role in defining the interaction between two different liquid compounds^44^, resulting in a great influence on the dissolving capacity of the main with the target liquid material. In the present case, the main compound is changing the spectral width of the LUMO associated transitions, which directly translates to changes in the intermolecular interaction habits of the liquid within the frontier orbital model. As the spectral width changes due to the different intra- and intermolecular conformations, these should subsequently influence the compound’s dissolving capacity of, e.g., water. According to Freire *et al*. the mole fraction solubility of water increases as the chain-length of perfluoroalkanes rises^2^. This coincides with the observations and conclusions drawn from the LUMO differences in the XA spectra of the presented materials leading to a direct impact of the different conformational geometries on the dissolving capacity of water in chain-shaped perfluorocarbons. We note that the conclusions drawn by Hamza *et al*. mention that the high amount of different molecular conformations in perfluorocarbons affect the dissolving capacity of liquids and gases, further supporting our findings^18^. To further understand this concept of conformational geometries influencing their capacity of dissolving other liquids and possibly gases, an extension to more complex perfluoro compounds and gas loaded samples, as well as molecular dynamics investigations, is recommended.

Theory predicts an additional small transition attributed to π_x_(C-C) resonances (see supplementary material for the molecular orbitals) at 292.2 eV, which creates a small shoulder on the low energy side of the resonance at 292.8 eV. The experimental spectra don’t give clear evidence for this transition, but the non-uniform energy changes on the lower energy side, compared to the higher energy side, give rise to a speculation regarding the existence of this transition. The form of the participating MO suggests an involvement with the conformation effect to a similar degree to the π_z_(C-C) resonance described above. Under these circumstances the transition would also be subject to the broadening effect explained earlier giving rise to a non-uniform energy change on the low energy side of the 292.8 eV resonance. Further analysis of this transition will be subject to future investigations as more data on different perfluoroalkanes is needed for an unequivocal understanding.

**Figure 2:**
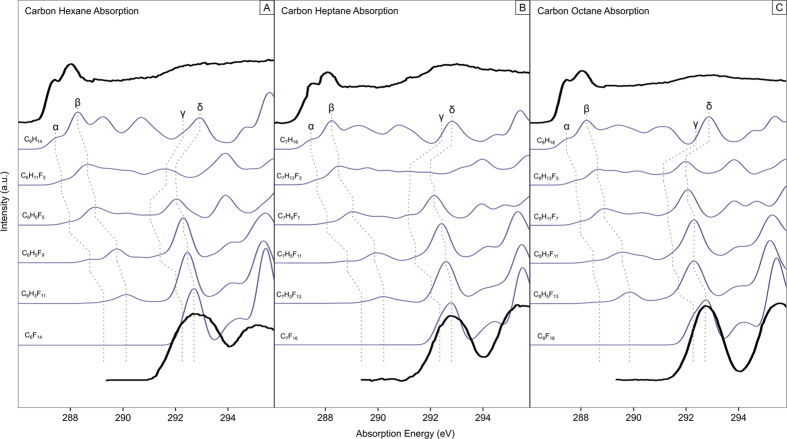
Experimental (black lines) and calculated (blue lines) carbon K-edge X-ray absorption spectra for hexane (**A**), heptane (**B**), octane (**C**) and their respective fluorinated compounds. Dashed lines indicate the shifts of the spectral features.

The experimental carbon K-edge XA spectra for the hydrocarbons and perfluorocarbons, the calculated spectra for the respective molecules as well as the stepwise fluorinated hydrocarbon derivatives are presented in Fig. 2. The figures also visualize the energy shifts of the four main features upon fluorination for the XA spectra. The dimension of the shifts gives rise to a possible direct σ- or π-character assignment for the participating orbitals in these features as described by Brundle *et al*. (2–4 eV corresponds to σ, 0–0.5 eV corresponds to π)^24^,^25^, where π is of out-of plane and diffuse (Rydberg-like) character, but not of anti-bonding character like π^*^. This information is summarized in Table 1.

The energy shifts reveal strong similarities between the main-features of each molecule, as suggested above. Additionally, a glance at the MO’s involved in each particular transition reveals significant similarities between the transitions of each molecule. Small differences exist, which can be attributed to the addition of (CF_2_)- or (CH_2_)-chains and the resulting small changes in molecular length, but the overall molecular character assignment is equivalent. Resonance α is the only not clearly assignable feature, but due to the strength of the energy shift a clear trend to σ-like character can be seen.

A closer look at features α and β reveals that they disappear and don’t have a counterpart in the spectra of the perfluorocarbons. In a previous work we found that the first two features of the ring-shaped decalin also disappear upon fluorination to perfluorodecalin (PFD)^23^. Through investigation of the X-ray emission (XE) spectra of both decalin and PFD the appearance of two features of similar character to the vanished ones was discovered in the XE region of PFD. From the surmised fluorination-dependent MO occupancy change from the unoccupied to the occupied MO-region we concluded a direct impact to the inertness of the perfluorinated molecule according to the frontier orbital model^23^,^44^. Under consideration of the missing XE data, we can hypothesize a similar effect occurring in the molecules reported in this work, but further investigation of the XE regime is needed to gain more insight into the nature of the orbital change and orbital character in these transitions.

**Figure 3:**
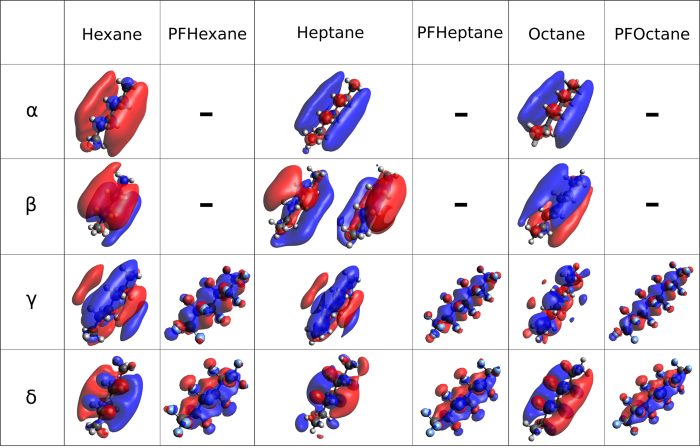
Table depicting the involved MO’s in each particular transition, sorted by the corresponding hydro- and perfluorocarbons.

Features γ and δ behave according to the theory of the perfluoro effect^24^ with a controlled stabilization of the MOs due to the fluorination (see Fig. 3). We note that the step from pure hydrocarbon to the first fluorinated hydrocarbon derivative involves a significant energy jump to the lower energy region. This energy shift is the result of the transformation of the previous pure carbon orbitals because of the substitution of hydrogen with electronegative fluorine. In case of γ and δ the involvement of fluorine quenches the MO, due to its shape, into a more confined space surrounding the carbon atoms. The first fluorination step therefore has the strongest impact on the MO, while the subsequently added fluorines stabilize the quenched MO, resulting in a stronger energy jump for the first fluorination. Additionally, the fluorine acts like a shield to shelter the MO from outside influence in a similar way to the previously reported PFD, where the orbitals are largely localized on the carbon atoms, which are repulsively interacting with a potential barrier created by the exchange interaction of the fluorine atoms^22^. This leads to a higher relative inertness within the understanding of the frontier orbital model and can be recognized as one of the reasons to the high relative inertness of perfluorinated materials. Subsequently, as a similar shielding effect was found for the presented samples, we conclude that one reason for the high relative inertness of chain-type perfluorinated materials is that the “shield” is inhibiting interactions. A general trend can be concluded for simple liquid ring- and chain-shaped perfluorinated materials and further research conducted on more complex perfluoro compounds is in progress to deliver a more thorough understanding of these findings.

## Discussion

Fundamental insights and a comprehensive picture of the electronic structure of hexane, heptane and octane as the parent molecules, as well as their respective perfluorinated counterparts, were presented based on XA spectroscopic data. In addition DFT calculations for the hydrocarbons, the stepwise fluorinated hydrocarbons and the perfluorocarbons were performed. Additional insights into the energy shift induced by fluorination, the so-called perfluoro effect, were drawn and these provided significant connections to previous works^22^,^23^. We observed changes in the electronic structure due to the conformation effect induced by the increase of (CH_2_)- and (CF_2_)-chains for the investigated molecules and detected that different conformational geometries mainly influence the chemical and electronic interaction between the molecules. Subsequently, that may be one of the main reasons for the slight differences in chemical and biological character of the presented liquid materials. Interestingly, the conformational geometries can greatly influence the LUMO of the perfluorocarbons leading to an impact on the dissolving capacity with other materials like water. As a full understanding of the gas dissolving capacity of perfluorocarbons was not found until this date, to our knowledge, a deeper investigation into this conformation effect and its extension to larger, more complex perfluoro systems and gas-loaded samples is of particular interest for medical applications like blood substitute formulations and liquid breathing^1^,^2^,^15^,^16^. We reported an indication for a MO alteration similar to the previously investigated ring-shaped perfluorodecalin and decalin delivering support to the validity of this concept and its possible extension to linear perfluoro systems. Further support for the previously reported fluorine “shielding” effect in perfluorocarbons^22^ was conveyed extending our understanding of this concept to the relative inertness of chain-shaped perfluorocarbons. In summary the validity of various concepts was confirmed and their extension to linear perfluoro systems presented a deeper insight into the basis of the relative inertness and the special properties of perfluoro compounds and their possible applications.

## Methods

### Experimental Details

Perfluorooctane (PFO), perfluoroheptane (PFHep), perfluorohexane (PFHex), octane, heptane and hexane were obtained from Sigma-Aldrich with purities ranged above 85% (>98% for PFO, >85% for PFHep, >99% for PFHex, >99% for Octane and Heptane, >95% for Hexane). XA spectra at the carbon K-edge were measured by using a transmission-type liquid flow-cell connected at the soft X-ray undulator beamline BL3U at the UVSOR-III Synchrotron^45^. The liquid cell consists of four regions, which are separated by three independent 100 nm thick Si_3_N_4_ membranes. Soft X-rays under vacuum (region I) pass through the buffer region filled with helium gas (region II) and the thin liquid layer (region III) and finally reach a photodiode detector in the last region filled with helium gas (region IV). The liquid sample is sandwiched between two Si_3_N_4_ membranes with pressed Teflon spacers set between the window frames of the membranes. By adjusting the helium pressure in the regions II and IV, the liquid layer thickness can be optimized between 2000 and 20 nm. Substitution of the liquid by other samples is performed in combination with a tubing pump system. Further details of the liquid cell are described elsewhere^46^,^47^. The energy resolution of incident soft X-rays at C K-edge is set to 0.2 eV. The energy calibration of the spectrometer was performed using absorption lines of CH_4_ gas which was mixed with He gas.

### Computational Details

The presented theoretical calculations were carried out with the ORCA program package^29^. Molecular geometry optimizations were performed using the B3LYP^48^,^49^ density functional method employing the def2-TZVP basis set^50^,^51^. During the optimization calculations, the resolution of identity approximation^52^,^53^,^54^,^55^,^56^ was used employing the def2-TZV/J basis set^57^. Transition energies and moments for the K-edges were calculated with time dependent DFT (TD-DFT). The core-hole excited state calculations are based on the computation of the dipole length and dipole velocity formalisms. Additionally, no intermolecular interaction effects in liquid phase are included. K-edge absorption spectra were obtained from the calculated transition moments by applying a Gaussian type broadening of 0.8 eV.

## Additional Information

**How to cite this article**: Brandenburg, T. *et al*. Impacts of Conformational Geometries in Fluorinated Alkanes. *Sci. Rep.* **6**, 31382; doi: 10.1038/srep31382 (2016).

## Supplementary Material

Supplementary Information

## Figures and Tables

**Table 1:** Energy shifts and derived MO character for the XA features.

feature	energy shift upon complete fluorination (PFHex) [eV]	energy shift upon complete fluorination (PFHep) [eV]	energy shift upon complete fluorination (PFO) [eV]	orbital character
α	1.76	1.68	1.70	σ-like
β	2.11	2.10	2.06	σ
γ	0.15	0.19	0.14	π_x_(C-C)
δ	0.17	0.09	0.10	π_z_(C-C)

Involved MO’s for the corresponding transitions are depicted in Fig. 3.
