# Supplementary material for: Impacts of Conformational Geometries in Fluorinated Alkanes
Source: Sci Rep. 2016 Aug 16;6:31382. doi: 10.1038/srep31382 (PMC4985654; doi:10.1038/srep31382)
Supplement: Supplementary Information [file srep31382-s1.doc]

Supporting Information

Impacts of Conformational Geometries in Fluorinated Alkanes

Tim Brandenburg,^a,b,*^ Ronny Golnak,^a,b^ Masanari Nagasaka,^c^ Kaan Atak,^a,b^ Sreeju Sreekantan Nair Lalithambika,^a,b^ Nobuhiro Kosugi,^c^ Emad F. Aziz ^a,b,c,**^

^a^ Institute of Methods for Material Development, Helmholtz-Zentrum Berlin für Materialien und Energie, Albert-Einstein-Straße 15, 12489 Berlin, Germany

^b^ Department of Physics, Freie Universität Berlin, Arnimallee 14, 14195 Berlin, Germany

^c^ Institute for Molecular Science, Myodaiji, Okazaki 444-8585, Japan

AUTHOR INFORMATION

Corresponding Author

* tim.brandenburg@helmholtz-berlin.de

** emad.aziz@helmholtz-berlin.de

**
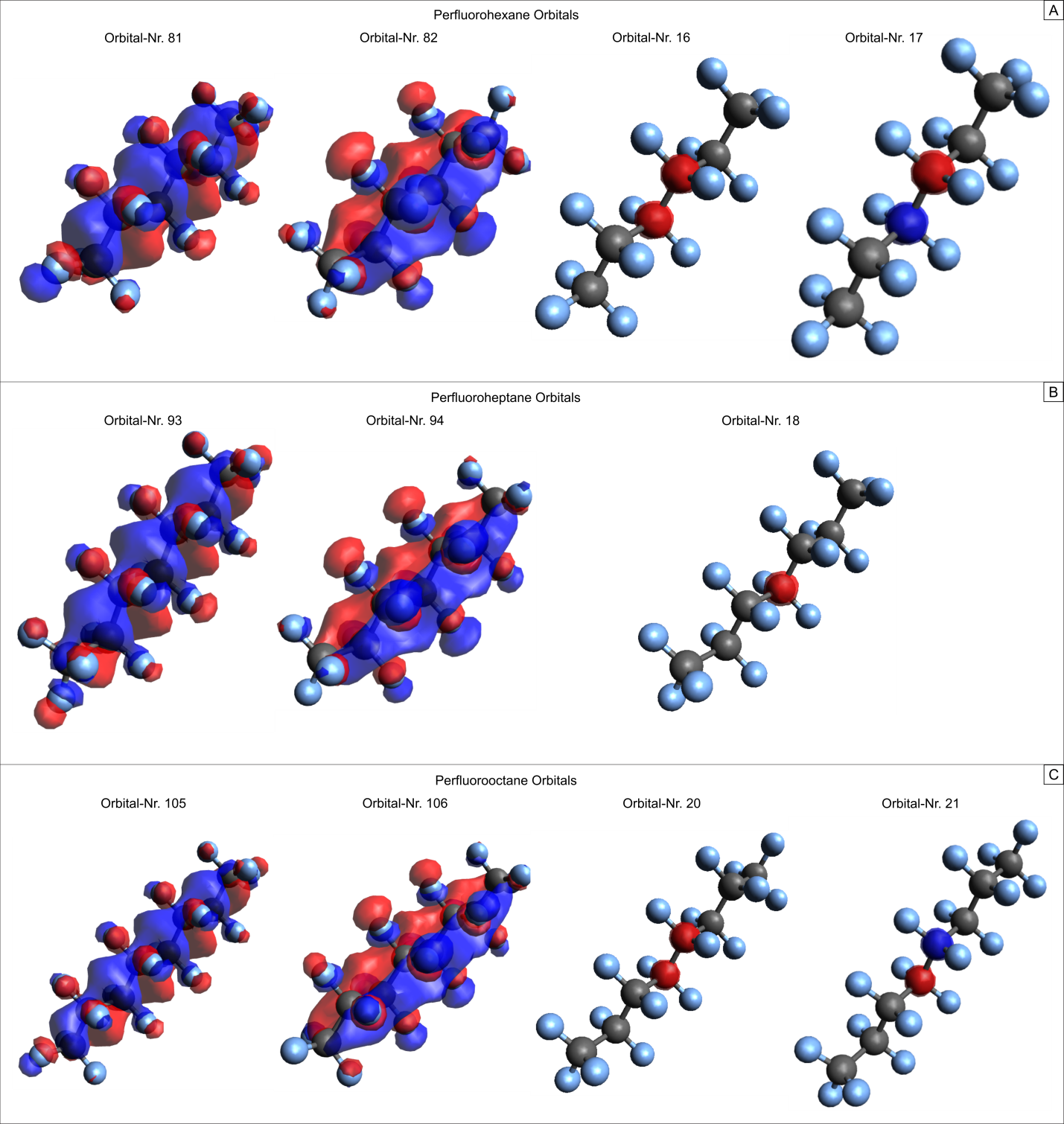
**

**Sup 1.** Molecular orbitals involved in the main resonance of the perfluorocarbon X-ray absorption spectra at 292.8 eV. Similar orbitals can be found for each molecule. The core orbitals represent the innermost carbon atoms (for even number of (CF_2_)-chains two carbons, for uneven number of (CF_2_)-chains one carbon). The unoccupied orbitals represent π_z_(C-C) and π_x_(C-C) resonances.


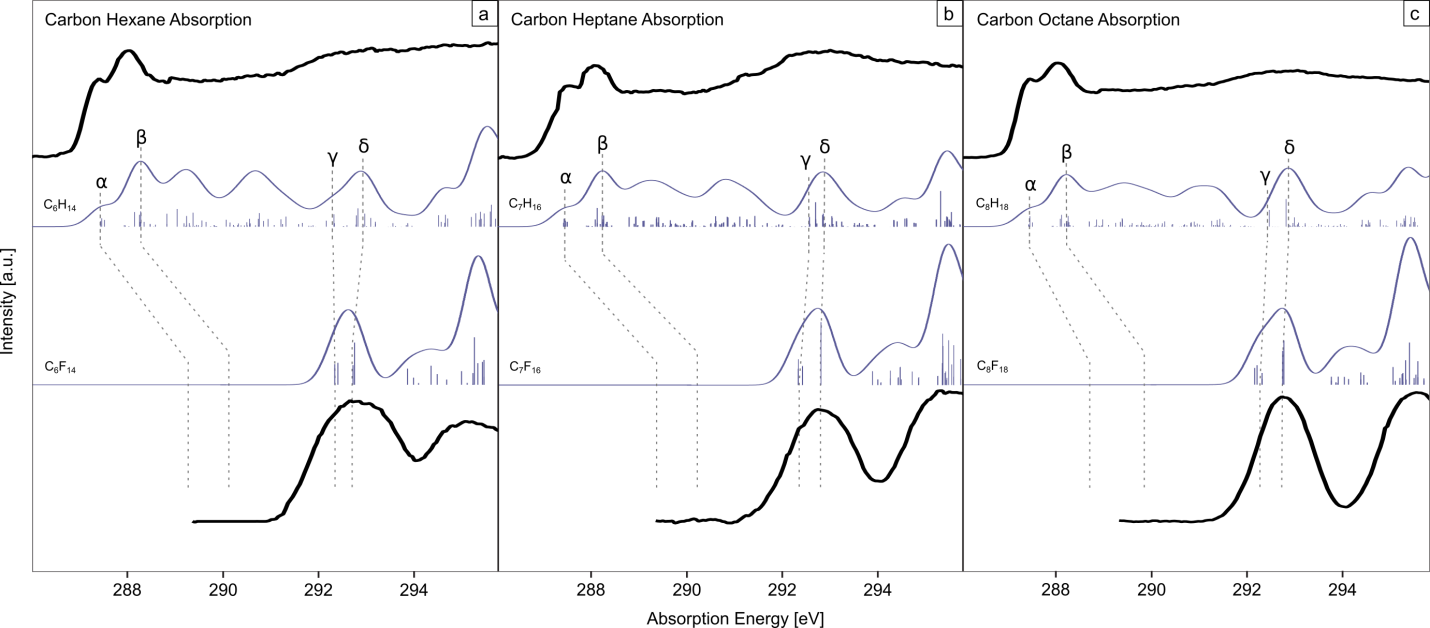


**Sup 2.** Experimental (black lines) and calculated (blue lines and transition bars) carbon K-edge X-ray absorption spectra for hexane (a), heptane (b), octane (c) and their respective fluorinated compounds. Dashed lines indicate the shifts of the spectral features.
